# Identification of Drought-Responsive MicroRNAs from Roots and Leaves of Alfalfa by High-Throughput Sequencing

**DOI:** 10.3390/genes8040119

**Published:** 2017-04-13

**Authors:** Yue Li, Liqiang Wan, Shuyi Bi, Xiufu Wan, Zhenyi Li, Jing Cao, Zongyong Tong, Hongyu Xu, Feng He, Xianglin Li

**Affiliations:** Institute of Animal Sciences, Chinese Academy of Agricultural Sciences, Beijing 100193, China; liyue_s@163.com (Y.L.); wanliqiang@caas.cn (L.W.); bsy9239@163.com (S.B.); xiufuwan@163.com (X.W.); lizhenyily@163.com (Z.L.); pandajing0919@163.com (J.C.); dradon.tong@163.com (Z.T.); xhy_rensheng@163.com (H.X.); hefeng@caas.cn (F.H.)

**Keywords:** alfalfa, drought, microRNA, small RNA, differential expression

## Abstract

Alfalfa, an important forage legume, is an ideal crop for sustainable agriculture and a potential crop for bioenergy resources. Drought, one of the most common environmental stresses, substantially affects plant growth, development, and productivity. MicroRNAs (miRNAs) are newly discovered gene expression regulators that have been linked to several plant stress responses. To elucidate the role of miRNAs in drought stress regulation of alfalfa, a high-throughput sequencing approach was used to analyze 12 small RNA libraries comprising of four samples, each with three biological replicates. From the 12 libraries, we identified 348 known miRNAs belonging to 80 miRNA families, and 281 novel miRNAs, using Mireap software. Eighteen known miRNAs in roots and 12 known miRNAs in leaves were screened as drought-responsive miRNAs. With the exception of miR319d and miR157a which were upregulated under drought stress, the expression pattern of drought-responsive miRNAs was different between roots and leaves in alfalfa. This is the first study that has identified miR3512, miR3630, miR5213, miR5294, miR5368 and miR6173 as drought-responsive miRNAs. Target transcripts of drought-responsive miRNAs were computationally predicted. All 447 target genes for the known miRNAs were predicted using an online tool. This study provides a significant insight on understanding drought-responsive mechanisms of alfalfa.

## 1. Introduction

Alfalfa (*Medicago sativa* L.) is an important forage species, with high nutritional quality and high yield [1]. As a legume plant, alfalfa is capable of fixing nitrogen to nitrate in nodules, by establishing a symbiotic relationship with *Rhizobium* in the root system, making it an ideal crop for sustainable agriculture. Thus, planting alfalfa can also improve the condition of the soil and reduce the usage of fertilizer. Alfalfa also has the potential to become a crop for bioenergy resources, with many appropriate attributes including high biomass yield potential [2,3]. However, the yield of alfalfa is often constrained by diverse abiotic stresses, including drought. Drought is a common environmental stress, affecting plants productivity [4,5]. Understanding the molecular mechanism of the alfalfa response to drought stress is a necessary step towards improving the drought tolerance of alfalfa.

Plants respond to stresses by regulating the expression of specific genes to avoid or minimize cellular damage, in order to adapt to stress conditions [6]. Gene regulation occurs at multiple levels, including transcriptional, post-transcriptional, and epigenetic levels. At the transcriptional level, many genes and transcriptional factors related to drought stress responses have been identified, including those involved with abscisic acid (ABA) regulation. Through the action of a number of known transcription factors, ABA-dependent and independent pathways can be induced by drought [7], which results in the activation of Late Embryogenesis-Dependent (LEA) protein synthesis, active oxygen scavenging enzymes, and osmolytes [8,9]. At the epigenetic level, evidence suggests that DNA methylation, histone modification and chromatin remodeling are related to regulating the plant response to drought [10,11,12].

Recently, the discovery of MicroRNAs (miRNAs) sheds light on post-transcriptional gene regulation. MiRNAs are 21 to 24 nt in length, and are noncoding small RNAs (sRNAs) that negatively regulate gene expression [5,13,14]. In plants, the biogenesis of miRNAs has been reviewed by several articles [5,13,14]. Briefly, the miRNA gene is transcribed to primary miRNAs by polymerase II; then the primary miRNA is 5′capped and 3′polyadenylated to form a classic stem-loop structure that is processed into a pre-miRNA by Dicer-Like Protein 1 (DCL1); subsequently, the pre-miRNA is cleaved into a double strand miRNA:miRNA* duplex by DCL1 in the nucleus, and the duplex is then separated into miRNA and miRNA* by helicase in cytoplasm. MiRNAs repress gene expression through guiding an RNA-induced silencing complex (RISC) to cleave target mRNAs, or inhibit translation of target mRNAs. Plant miRNAs are highly complementary to target mRNAs, and miRNA sequences are usually conserved in related organisms. Unlike miRNAs, small interfering RNAs (siRNAs) are double-stranded RNA, and are processed from double-stranded precursors [15,16]. MiRNAs as well as siRNAs can be incorporated into RISC, thus repressing mRNA expression [17].

It has now been accepted that miRNAs play vital roles on multiple crucial biological and metabolic processes in plants. For example, leaf morphology was severely affected by miR319 overexpression in the *jaw-D* mutant [18]. Changing the expression level of miR172 can affect flower morphology [19]. A group of miRNAs also play pivotal regulatory roles in the plant response to various stresses, such as hypoxia [20], salinity [21], cold [22], UV-B radiation [23], nutrient deficiency [24], heavy metal [25], and drought [4,26].

Studies from sequencing, microarray, quantitative reverse transcription polymerase chain reaction (qRT-PCR) and Northern blot have demonstrated that the expression level of miRNAs was altered by drought in many plant species, including *Medicago truncatula* [27], *Vigna unguiculata* [28], *Manihot esculenta* [29], *Solanum tuberosum* [30], *Gossypium* [31], *Nicotiana tabacum* [32], *populous* [33], *Oryza sativa* [6], *Saccharum officinarum* [34], *Panicum virgatum* [35], *Hordeum vulgare* [36], *Triticum turgidum* L. ssp. *durum* [37,38], and foxtail millet [39]. Importantly, genes associated with the miRNA pathway, such as *DCL1* and *ARGONAUTE* (*AGO*) genes, were up-regulated under drought stress, implying the involvement of miRNA in plant adaptation to drought [40]. Conversely, changing the expression level of miRNAs can affect plants’ response to drought stress [41]. All these studies suggest that miRNAs are a potentially powerful tool for modifying drought resistance in alfalfa and other legumes.

In alfalfa, only salinity-regulated and fall dormancy-related miRNAs have been identified so far, both using the publicly available genome of *Medicago truncatula* [1,21]. In 2014, Fan et al. identified some fall dormancy-related miRNAs in two varieties of alfalfa (Maverick and CUF101). In 2015, Long et al. identified many known miRNAs, along with 68 miRNA candidates [21]. However, none of the previous studies have explored drought-responsive miRNAs. Identifying these drought responsive miRNAs is valuable for investigating miRNA-mediated gene regulation in alfalfa. In this context, the goals of our work are: (i) to identify target miRNAs that may regulate stress responses to drought in alfalfa, and explore the underlying mechanisms for miRNA function in the drought stress response of alfalfa; and (ii) to discover novel miRNAs in alfalfa. In this study, 12 sRNAs libraries from the leaves and roots of alfalfa plants in response to control or drought conditions were established and sequenced with the high throughput sequencing Hiseq2500 platform. The data set of 12 sRNAs libraries from alfalfa was analyzed in silico. We identified 348 known miRNAs and predicted 281 novel miRNAs in alfalfa. MiRNA qRT-PCR was also adopted for validation of the expression of selected miRNAs, which was examined by high throughput sequencing. Furthermore, characterization of target genes was performed by using bioinformatic approaches. Through high-throughput sequencing and bioinformatics analysis, known drought stress-responsive miRNAs and miRNA candidates in alfalfa were identified. This study will be very helpful for understanding post-transcriptional regulation under drought stress in alfalfa, and improving the drought tolerance of alfalfa and other legumes.

## 2. Materials and Methods

### 2.1. Plant Materials and Experiment Design

*Medicago sativa* L. cv. Aohan was used in this study. This cultivar was kindly provided by Dr. Liqiang Wan (Institute of Animal Sciences, Chinese Academy of Agricultural Sciences, Beijing, China). Alfalfa plants were grown in pots with a diameter of three inches, containing a mixture of sand:vermiculite (1:1 *v/v*) in a growth chamber at 25–28 °C under a 16 h light/8 h dark photoperiod. Plants were supplied daily with Murashige-Skoog (MS) nutrient solution. Alfalfa plants at the age of eight weeks were then randomly separated into two groups, namely drought treatment and control groups. Drought stress treatment was imposed by withholding water supply for 10 days. The control plants received normal watering throughout the experiment. The volumetric water content of soil (VWCS) was detected before sampling by using a WET Sensor (Delta-T Devices Ltd., Cambridge, UK) which is a soil moisture sensor. The VWCS of control group was approximately 45%, and the VWCS of treatment group at the fifth day was 26.7%, at the tenth day this was 16.8% in the stress treatment.

### 2.2. Total RNA Isolation

Root and leaf samples from both drought and control plants were collected at the fifth and tenth days during the stress treatment, respectively. For each sample of leaf or root, three biological replicates were prepared, with each biological replicate collected from 10 plants. Samples were fast frozen in liquid nitrogen, stored at −80 °C.

Total RNA samples were extracted and then were prepared for sequencing, reverse transcription PCR and qRT-PCR. Equal quantities of RNA isolated from leaves and/or roots at each stress stage were pooled, using HiPure Plant RNA Mini Kit (Magen, Shanghai, China) according to the manufacturer’s instructions.

### 2.3. Small RNA Library Construction and High-Throughput Sequencing

A total of four groups of RNA samples (WL: leaves with watering, WR: roots with watering, DL: leaves with drought stress, and DR: roots with drought stress), each with three biological replicates, were prepared. For each group, RNA at both five days and 10 days were equally pooled to make one sample. Thus, a total of 12 samples were used to construct sRNA libraries. Since we pool samples at different stress treatment time points (five days and 10 days), only robust and consistent responses could be detected.

Total RNA was isolated by 15% polyacrylamide gel electrophoresis, and RNA molecules that were less than 50 nt in length were enriched and ligated with proprietary adapters. The RNA samples ligated with adapters were reverse-transcribed and amplified by PCR to produce sequencing libraries. The 12 sRNA libraries from alfalfa leaves and roots were sequenced on an Illumina Hiseq 2500 (Santiago, CA, USA) platform at the Guangzhou RiboBio Company, China. The raw data has been deposited in the Sequence Read Archive of NCBI, with a SRA data study accession number of SRP094823.

### 2.4. Identification of Known and Novel MicroRNAs

The raw sequencing reads were processed to obtain unique sequences and read count/unique reads as per the procedure reported by Hackenberg et al. [36]. First, sRNA reads of 17–45 nt were annotated to Rfam databases (Rfam 11.0, rfam.janelia.org) [42], to identify and eliminate transfer RNA (tRNA), ribosomal RNA (rRNA), small nuclear RNA (snRNA) and small nucleolar RNA (snoRNA) sequences from the sRNA reads. Then we computed the rest of sequences for sequence redundancy, and mapped these sequences to miRBase (release 21, http: //www.mirbase.org/) [43] without mismatches to identify known miRNAs. After removal of the known miRNAs, the remaining sequences were used to predict the novel miRNAs. The unique sequences were mapped to the *M*. *truncatula* genome version 4.0 (http://www.medicagohapmap.org/?genome) using Burrows-Wheeler Alignment (BWA) [44] to get pre-miRNA sequences for prediction of novel miRNAs.

Novel miRNAs were predicted by using Mireap [45]. Novel miRNA candidates were identified according to the criteria reported by [46]. The normalization of reads count and calculation of log Fold change were processed as [21] described.

### 2.5. MicroRNA Validation by qRT-PCR

The same RNA samples used for Illumina sequencing were employed in qRT-PCR analysis. Total miRNA was reverse transcribed to complementary DNA (cDNA) using the miRcute miRNA First-Strand cDNA Synthesis Kit (Tiangen, Beijing, China). According to the manufacturers’ instructions, the miRNAs were polyadenylated and reverse transcribed in one step using miRNA RT Enzyme Mix (*E. coli* Poly(A) Polymerase, RTase and RNasin). The Universal RT Primer was provided in the kit. Then the first-strand cDNA was prepared for qRT-PCR analysis.

qRT-PCR was performed on an Applied Biosystems 7300 Real-Time PCR System (Applied Biosystems, Foster City, CA, USA). The reaction system was constructed according using the miRcute miRNA qRT-PCR Kit (Tiangen, Beijing, China) containing SYBR^®^ Green detection reagents (Applied Biosystems). The cycling parameters were set according to manufacturers’ recommendations. Briefly, the cycling parameters were: initial polymerase activation step for 15 min at 95 °C, 40 cycles for 20 s at 94 °C for denaturation, 34 s at 60 °C for annealing and elongation, followed by a disassociation stage. The forward primers were designed according to the miRNA sequences of interest and synthesized by Invitrogen (Carlsbad, CA, USA). The sequences of the forward primers are supplied in Table S8. The melting curves of the PCR products can be found in Figure S1. The Universal qPCR Primer was provided in the kit. The transcript abundance of each miRNA was normalized to U6 snRNA, and the 2^−ΔΔCt^ method was used to calculate relative expression of miRNAs [47]. In order to compare pair-wise differences in expression, a Student’s *t*-test was performed by using Statistical Analysis Software (SAS) program.

### 2.6. MicroRNA Target Prediction and Function Analysis

Target genes of drought-responsive miRNAs in alfalfa were predicted using the psRNATarget online tool (http://plantgrn.noble.org/psRNATarget/). Gene annotation can also be accomplished by using this online tool. psRNATarget is a modified version of miRU. The *M*. *truncatula* spliced transcript sequences 4.0 V1 was selected as the transcript library for target search. Mature miRNA sequences responsive to drought and identified in alfalfa roots and leaves, were used as custom miRNA sequences. Default parameters for target prediction were used.

## 3. Results

### 3.1. Overview of Small RNAs from Alfalfa via High-Throughput Sequencing

**Figure 1:**
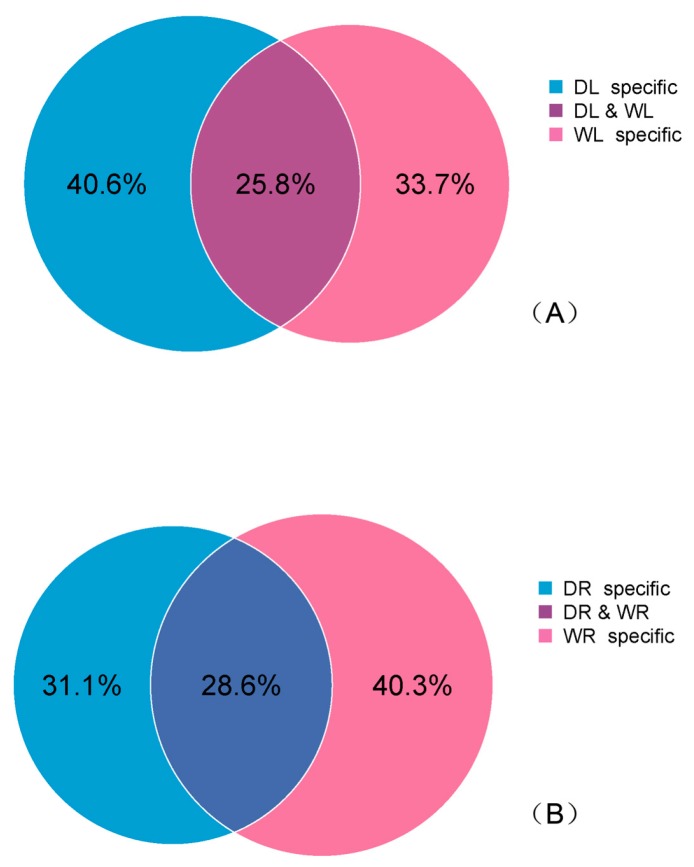
Venn diagram illustrating common and specific smallRNA (sRNA) sequences induced by drought stress. (**A**) Common and specific sRNA sequences in leaves; (**B**) Common and specific sRNA sequences in roots.

A total of 12 sRNA libraries comprising of four samples (WL: leaves with watering, WR: roots with watering, DL: leaves with drought stress, and DR: roots with drought stress) were generated using the Illumina HiSeq 2500 platform, each with three biological replicates. In order to obtain high quality data sets, adaptors and low-quantity reads were removed, and 12 million to 16 million clean reads at 17–45 nt in length were obtained from each of the 12 libraries. The details of raw reads and clean reads for each library are shown in Table 1. We analyzed common/specific sequences between four groups (WL, DL, WR and DR) for the total sRNA sequences. There were 40.6% and 31.1% specific sequences in group DL and DR, respectively (Figure 1), which indicates that there are changes occurring on the molecular level. These changes may be caused by drought stress, or by other phenomena such as plant development.

**Figure 2:**
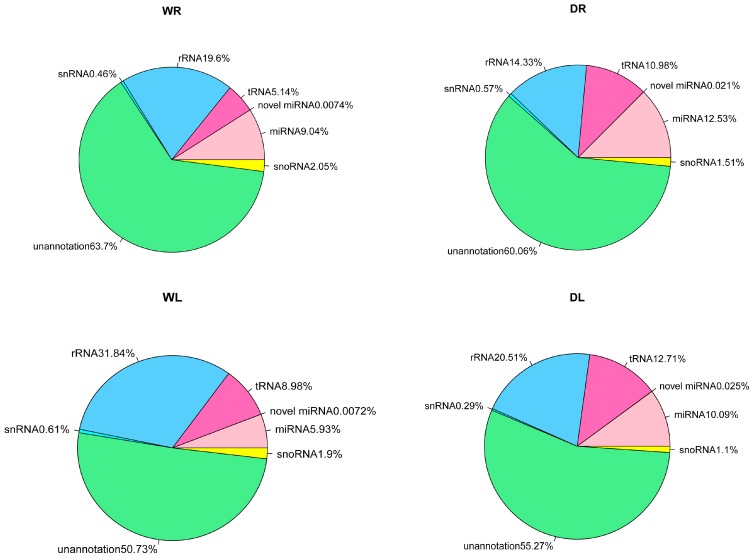
An overview of the frequency of different sRNA species present in the different groups. snoRNA: small nucleolar RNA; miRNA: microRNA; tRNA: transfer RNA; rRNA: ribosomal RNA; snRNA: small nuclear RNA.

In order to classify the sRNA sequenced reads into different categories and identify all the miRNA sequences in the 12 libraries, we mapped the reads to specific databases. Generally, the sequenced sRNA reads mapped to the miRBase database were abundant in drought samples (DL 10.09%; DR 12.53%) compared to control samples (WL 5.93%; WR 9.04%). The percentage of the sRNA reads mapped to the tRNA database was greater in drought samples (leaves 12.71%; roots 10.98%) than in control samples (leaves 8.98%; roots 5.14%). For rRNA, the opposite was true. Both drought and control samples had large numbers of unannotated reads (WR 63.71%, WL 50.74%, DR 60.08%, DL 55.3%) (Figure 2). The number of total sequences that matched *M*. *truncatula* genome is given in Table 1. Sequences failing to map to the genome ranged from 5.5% to 17.3%, with exception of WR (30.9%) and DR (40.1%). These unmapped reads may have been due to unavailable genome or sequencing errors.

### 3.2. Identification of Known miRNAs

Known miRNAs in alfalfa were identified by mapping sRNA sequences generated from each library to the miRNAs database (miRBase 21, released in June 2014). After a homology search and removal of miRNAs with expression levels less than 10, 287, 314, 204 and 142 miRNAs were identified from the groups WL, DL, WR and DR, respectively. There were 348 known miRNAs belonging to 80 miRNA families that were identified from the 12 libraries. The details of miRNAs of each library are listed in Tables S1 and S2. Among these miRNA families, the miR159 and miR166 families had the most reads, with exception of the replicate WR1 (miR166 and miR398 families). Of these identified miRNAs, miR166 contained the most members, including miR166a-g, miR166i and miR166u. Additionally, the most abundant miRNA was miR5213-5p followed by miR166a-3p in the DR1, DR2 and DR3 libraries. In the other libraries, miR166a-3p was the most abundant, followed by miR5213-5p or miR159 (see Tables S1 and S2).

### 3.3. Identification of Novel miRNAs

**Figure 3:**
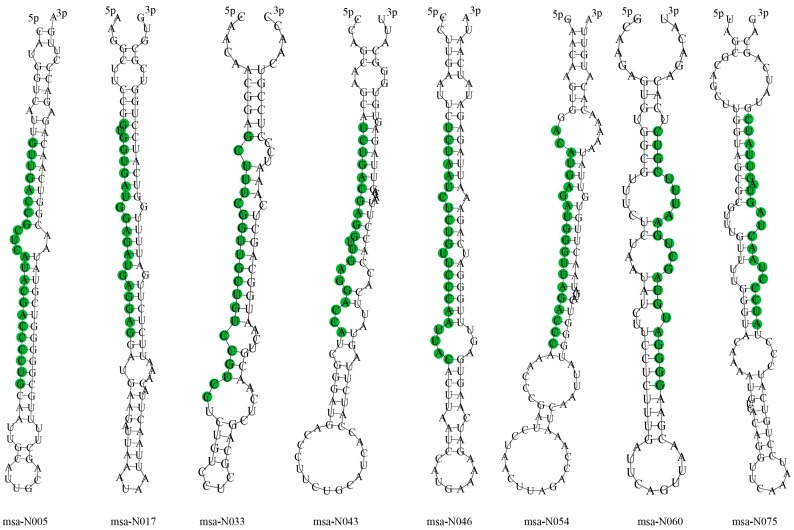
Representatives of precursor hairpin structures for predicted miRNAs from the *M. truncatula* genome database. Mature miRNA sequences are shown in green.

A total of 281 novel miRNAs were identified from the 12 sRNA libraries using Mireap software. For each library, detailed information of predicted novel miRNAs are listed in Table S3, including novel miRNAs sequences, reads length, reads number, GC contents, pre-miRNA sequences, miRNAs loci and pre-miRNA length. The read counts of these novel miRNAs ranged from 10 to 902. A total of 26 out of 281 novel miRNAs were sequenced over 100 times, while only six novel miRNAs were sequenced over 500 times. Eight novel miRNAs were found to exist in at least five libraries (Table 2), indicating that they are miRNA candidates with a higher level of confidence. Their precursor sequences, as well as the stem-loop hairpin secondary structure are shown in Figure 3.

### 3.4. Drought-Responsive miRNAs Identified in Alfalfa

To identify drought-responsive miRNAs, miRNAs which absent from two of three biological replicates were filtered out, then the normalized expression profiles of known miRNAs in drought-treated samples were compared to the control samples using generalized linear model analysis with the edgeR package [48]. A log Fold Change (logFC) change cut-off of 1 and a *p*-value ≤ 0.05 were used to obtain the differentially expressed miRNAs [49]. These differentially expressed miRNAs between the control and drought treatment group were called as “Drought-responsive miRNAs”.

**Figure 4:**
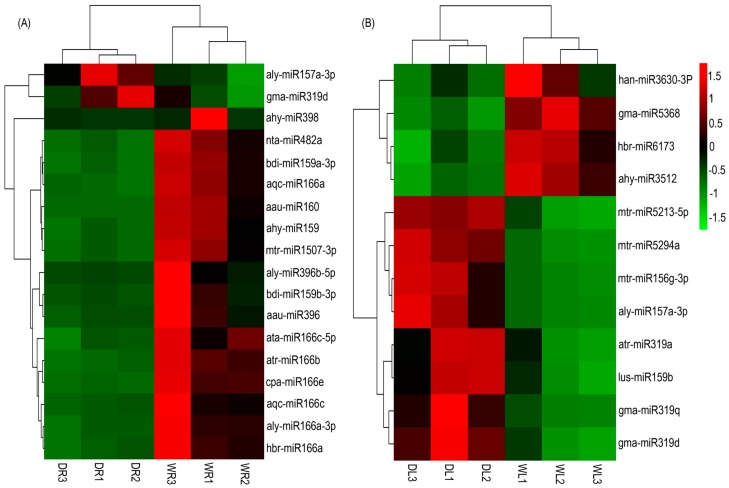
Heatmap representing the expression profile of the drought-responsive miRNAs. Differential expression of drought-responsive miRNAs in leaves (**A**) and roots (**B**). The upregulated miRNAs are showed in red, whereas the downregulated miRNAs are showed in green.

A total of 12 and 18 miRNAs were observed responsive to drought treatment in alfalfa leaves and roots, respectively (Figure 4). Some of drought-responsive miRNAs reported by previous studies were also detected in our analysis. For example, miRNAs miR166 and miR398 were found to be down-regulated in alfalfa roots, while miR319 and miR157 were up-regulated in both roots and leaves. Detail information of the drought-responsive miRNAs such as log fold-change, counts per million, and p-value, are given in Tables S4 and S5. MiR396, miR159/319, miR160, miR482, miR157 and miR1507 in alfalfa roots were also found to be drought-responsive. MiR156, miR3512, miR5368, miR3630, miR6173, miR5213 and miR5294 were drought-responsive in alfalfa leaves.

Our results also revealed that miR396, miR159, miR160, miR482, and miR1507 were down-regulated in alfalfa roots; miR3512, miR5368, miR3630 and miR6173 were down-regulated in leaves, whereas miR156, miR157, miR159/319, miR5213 and miR5294 were up-regulated in alfalfa leaves. The expression level of some miRNAs showed inconsistency and very high variation across replications. These miRNAs may have been be falsely identified by software, as the algorithm for discovering differential expressed miRNAs is subject to false positives. Validation of drought–responsive miRNAs by qRT-PCR experiments was therefore necessary. It is worthy to note that the expression of the homologues miRNAs belonging to the same miRNA family was consistently similar. For instance, in alfalfa roots, aqc-miR166a, hbr-miR166a and aly-miR166a-3p were all down-regulated.

We also detected other drought related miRNAs, but the expression levels of these miRNAs did not show significant changes, such as miR168, miR393, miR408 and miR2118. One of the reasons may have been due to the approach of sample pooling used in this study. The expression level of these miRNAs may have changed significantly at 5 days and/or 10 days, but we could not detect such changes in this experiment, because the significant change of expression level at one time-point could be canceled out by the expression level at another time-point after pooling the samples at five days and 10 days together.

### 3.5. Validation of Drought-Responsive miRNAs by qRT-PCR

To experimentally validate a number of selected miRNAs detected from the Illumina high-throughput sequencing, qRT-PCR technique was employed. Fifteen known miRNAs were tested by qPCR, and the results suggested that ahy-miR398, aau-miR396, mtr-miR1507-3p, aqc-miR166a, aly-miR166a-3p, ahy-miR3512, han-miR3630-3p and mtr-miR156g-3p showed similar expression patterns to those revealed by high-throughput sequencing analysis. However, we found that the expression level of aly-miR396b-5p as detected by qRT-PCR was inconsistent with that of our sequencing results, and the *p*-values of gma-miR319d, bdi-miR159a-3p, nta-miR482a, gma-miR5368 and mtr-miR5294a from qRT-PCR analysis showed discrepancies to the high-throughput sequencing data, these may have been caused by sequencing error or other reasons. The 15 known miRNAs are shown in Table 3. The qRT-PCR results suggest that our sequencing data are credible.

### 3.6. Targets of Drought-Responsive miRNAs and Their Functional Analysis

The mature sequences of the 18 miRNAs in roots and 12 miRNAs in leaves that were modulated by drought, were used to search for their targets in alfalfa (Tables S4 and S5). Target genes of some of the miRNAs identified in this study are already known, such as miR166, miR159/319, miR160, miR396, miR398, miR482, mir156 and miR157. As for the drought responsive miRNAs of which targets are unknown, we used the online tool psRNATarget to predict their targets, by matching the miRNAs to Mt 4.0, and the results are shown in Table 4.

A total of 445 target genes for the drought-responsive miRNAs, and 196 target genes for the eight novel miRNAs were predicted, and detailed information of the target genes including target ID and functional annotation are shown in Tables S6 and S7. We noted that most of these miRNAs had more than one predicted targets. Some miRNAs had no predicted target due to lack of genome information. A number of these predicted targets were involved in metabolism, growth and response to stresses. Some predicted targets play vital roles in abiotic stress responses. For instance, ahy-miR3512 was predicted to target three genes (Table 4), one of which was *spermidine synthase* (Medtr8g063940.1), which is involved in growth and resistance to adverse stresses including heat, salinity and drought.

## 4. Discussion

In this study, we constructed 12 libraries from different tissues of alfalfa treated with drought stress and well watering (control). All libraries were sequenced using the Illumina Hiseq 2500 platform. Small RNA deep sequencing from leaves and roots of alfalfa and comprehensive and systematic analysis were performed. We first identified drought-responsive miRNAs and their targets, and subsequently focused on discovering novel miRNAs.

In general, an RNAseq tag density of 2–5 million reads is sufficient for miRNA expression profiling and discovery applications [49]. In the current study, approximately 14–18 million reads for each library were generated by high-throughput sequencing. Furthermore, we sequenced three biological replicates for WL, DL, WR and DR. Our ur sRNA sequencing depth is sufficient not only for profiling of the miRNAs expression, but also for the discovery of poorly-expressed novel miRNAs.

Approximately 82%–94% of the reads sequenced in alfalfa leaves mapped to the *M. truncatula* genome, indicating that most of the miRNAs of *M. truncatula* and *M. sativa* are identical in leaves. However, only 58%–73% of the reads sequenced in alfalfa roots matched to the *M. truncatula*. This is similar to several previous studies, on peach [50] and on *M. truncatula* [25]. Known miRNAs comprised 5.1%–12.9% of the mapped reads, with DL and DR having higher proportions of known miRNAs (10.1%–12.5%) while WL and WR had a lower frequency (5.9%–9.0%). This indicates that the drought treatment activated some miRNA-related pathways. Unannotated sequences ranged from 50.7%–63.7%, which strongly implies the existence of large amounts of undiscovered sRNAs in alfalfa.

By In silico analysis, we identified 348 known miRNAs from the 12 libraries. The miR166, miR159, miR482 and miR2118 families were abundantly expressed. MiR166a-3p and miR5213-5p were the most abundant miRNAs. These highly expressed miRNAs may play important regulatory roles in gene expression. For example, miR166 is involved in plant organism morphogenesis, such as shoot apical meristem and floral development [51].

Discovering novel miRNAs is one of the advantages of next-generation sequencing compared to other technologies such as microarrays. In this study, 281 new miRNA candidates were predicted by computational methods. However, only a few miRNAs were commonly expressed in roots and/or leaves. Most of the novel miRNAs were uniquely expressed in each library, thus we cannot profile the differential expression of the novel miRNAs among libraries. For profiling the expression of the novel miRNAs, enhanced sequencing depth may be required. Moreover, the expression level of the predicted miRNA candidates was relatively lower than conserved miRNAs, and this result is in agreement with previous reports [25,50,52,53]. One possible explanation for this result is that the conserved miRNAs regulate target genes which may be involved in many important metabolic processes in Viridiplantae, while the expression of nonconserved miRNAs may be environmentally inducible or tissue-specific, thus the expression level of conserved miRNAs may be higher than non-conserved miRNAs [52,53].

Besides identifying known and novel miRNAs, high-throughput sequencing technology also provides an alternative way for evaluating the expression of miRNA genes. To obtain robust and consistently-expressed drought-responsive miRNAs, we pooled the samples from different time-points of stress together when constructing sRNA libraries according to [25,32,54,55]. To discover drought-responsive miRNAs from the sequencing data, the mature miRNA expression profile of drought-treated leaves and roots was compared with the control group, to identify miRNAs significantly modulated by drought. Finally, 18 known miRNAs in roots and 12 known miRNAs in leaves were screened as drought-responsive miRNAs.

In roots, 16 out of 18 drought-responsive miRNAs belonging to miR396, miR159, miR160, miR482, miR1507, miR166, miR156 and miR398 families were down-regulated. Since miRNAs negatively regulate their target genes, it can be predicted that targets of down-regulated miRNAs during drought stress may play positive roles for drought stress responses. Concurring with expectations, miR166, which down-regulates HD-ZIPIII transcription factors, was down-regulated. This indicates that the target gene of miR166 was up-regulated. HD-ZIPIII transcription factors are important for lateral leaf development, root development and axillary meristem initiation [56,57]. In *Triticum dicoccoides* [58] and in barley [59], miR166 was down-regulated under drought stress. Inversely, miR166 was up-regulated by drought in *M. truncatula* [60], which was different from our results. Similarly, Long et al. [21] previously reported that miRNAs in *M. truncatula* and *M. sativa* presented the opposite expression pattern under salt stress. The difference of expression pattern between *M. truncatula* and *M. sativa* may be caused by their different resistances to stresses, or the fact that the sRNA libraries were pooled from different time-points, which would have complicated the results.

MiR159/319 was also down-regulated in alfalfa root. The MYB family and TCP family are targets families of this miRNA, and both of them were identified by 5′ RACE [18]. Under drought conditions, most active MYB transcription factors are involved in ABA signaling pathway. Previous studies in *Arabidopsis* spp. indicate that some *MYBs*, including *MYB2*, are positive regulators of ABA signaling [61,62]. With the decreased expression of miR159 resulting in the positive regulation of the ABA signaling pathway, down-stream of ABA signaling pathways are stimulated, including root development. Similarly, miR160, which targets auxin response factors (ARFs), also has positive regulatory roles in drought stress responses [63].

Known targets of miR398 are involved in respiration and oxidative stress [64]. We found that drought stress down-regulated miR398 in alfalfa roots, in accordance with the results in maize [65] and *M. truncatula* [27]. However, Trindade et al. [60] found the opposite in *M. truncatula*. The differences in the expression of miR398 showed here may be caused by differences in species responses, duration of drought stress, and the metabolic states of the individual plants in different studies [5].

MiR396, miR482 and miR1507 were down-regulated under drought stress. However, their currently identified roles only include development or disease resistance; therefore, it can be predicted that they may have additional targets that are yet to be identified.

Two of these drought-responsive miRNAs (gam-miR319d and aly-miR157a-3p) were up-regulated in alfalfa roots. In order to conserve water and protect the cell, miRNAs are expected to be up-regulated during drought stress, so that those processes involved in normal growth and metabolism can be shut down. However, we failed to obtain valuable information about stress resistance from their target gene annotation. Interestingly, as a member of miR159 family, gam-miR319d was expected to be down-regulated by drought, to cause the positive regulation of the ABA signaling pathway, but it did not show down-regulation in our study. However, the target gene of gam-miR319d, was the NB-ARC domain protein, a disease resistance protein, instead of the MYB transcription factor, as predicted using the online program psRNATarget. There are two possible reasons to explain this result: (1) gam-miR319d may have other unknown targets that play negative roles in drought adaption in alfalfa; (2) gam-miR319d plays no role in drought adaption, but can be stimulated by drought through an unknown mechanism. With the function of gam-miR319d still being unclear, further studies will be needed to explore the roles of gam-miR319d in drought stress.

In alfalfa leaves, most of the drought-responsive miRNAs were involved in development, substance synthesis and transport. MiR3512, miR5368, miR3630-3p and miR6137, whose targets remain unknown, were down-regulated in this experiment. Target genes of miR3512, miR3630-3p and miR6137 were obtained using the online program psRNATarget. These targets include zein-binding protein, polyol/monosaccharide transporter, spermidine synthase, cytochrome P450 family 71 protein and TCP family transcription factor.

MiR159/319 is down-regulated in roots and up-regulated in leaves, which may indicate that the same miRNA could play different roles in different tissues. In *Arabidopsis* spp., *MYB33* and *MYB101* transcripts are targets of miR159a [66,67]. Under drought conditions, *MYB33* and *MYB101* could modulate stomatal movement by regulating the ABA signal, suggesting that miR159/319 plays a positive role on drought response by decreasing stomatal conductance. Additionally, miR156, miR157, miR5213 and miR5294 were up-regulated significantly (*p* < 0.01), and their targets were involved in development or disease resistance, indicating these miRNAs play some roles under drought stress.

In summary, 348 known miRNAs have been identified from alfalfa leaves and roots, and a number of candidates for drought-responsive miRNAs have been identified, thus this attempt paves the path for better understanding the drought-responsive mechanisms of alfalfa. In addition, the identification of 300 novel miRNAs also provides promising resources for future research in understanding post-transcriptional regulation in alfalfa. Future studies should focus on the verification of novel miRNAs and their predicted targets by experimental approaches, and additionally, the effects of drought-responsive miRNAs on drought tolerance need to be illuminated. To our knowledge, our study is the first systematic and comprehensive identification of drought-responsive miRNAs in an alfalfa species. This study is valuable for improving drought tolerance and systems to mitigate crop losses under drought stress.

## Figures and Tables

**Table 1:** Alfalfa small RNA (sRNA) sequencing datasets. Statistics of sRNA sequences for water and drought stress libraries from *Medicago sativa* leaf and root.

Library	Replicates	Raw Reads	Clean Reads	Reads Mapped to the Genome	Match Known miRNAs
WL	WL1	16,729,829	14,718,375	13,037,667	1,030,262
	WL2	18,529,495	16,118,371	15,226,670	917,066
	WL3	18,683,242	16,026,900	15,129,492	816,007
WR	WR1	17,888,348	15,351,268	11,164,625	1,360,347
	WR2	18,777,136	15,724,912	11,131,597	1,225,474
	WR3	17,354,872	14,048,339	8,964,877	1,472,074
DL	DL1	15,668,523	12,648,491	10,760,915	1,315,694
	DL2	14,266,305	12,378,478	10,236,124	1,269,855
	DL3	14,741,343	12,407,912	10,797,039	1,191,355
DR	DR1	16,484,366	14,594,872	8,586,263	1,814,906
	DR2	14,587,651	12,844,508	8,055,680	1,662,649
	DR3	14,256,856	12,721,466	7,391,569	1,554,103

Notes: WL: leaves with watering, WR: roots with watering, DL: leaves with drought stress, and DR: roots with drought stress.

**Table 2:** Eight novel miRNAs identified from *M. truncatula* genome databases.

Name	Sequences	Length	GC Contents	Loci	Minimum Free Energy (kcal/mol)
msa-N005	GUUGACCGCUCAUACGACCCCUG	23	60.87	NC_016411.2:1711404:1711490:+	−54.00
msa-N017	GUGGUGAUGGAGAUGAGGAG	20	55	NC_016410.2:44927035:44927123:−	−31.90
msa-N033	GCUUUCGGUUGCUGUCCGUCC	21	61.90	NC_016407.2:37807782:37807862:−	−22.80
msa-N043	UCUGACGAGGUUGAGGACCA	20	55	NC_016412.2:20603621:20603715:+	−22.30
msa-N046	UCUAAUCUCUGUUCCCAAUUAC	22	36.36	NC_016411.2:42295745:42295832:−	−33.90
msa-N054	ACAUGAGAUGGGUUAGACCC	20	50.00	NC_016414.2:14441574:14441672:−	−20.70
msa-N060	GGGGAUGUAGCUGAAUUUCGUC	22	50.00	NC_016408.2:4907664:4907746:−	−20.50
msa-N075	AUCCCUAACUAGUAGUUAUC	20	35.00	NC_016414.2:17652459:17652554:+	−20.30

**Table 3:** Expression levels detected by high-throughput sequencing and quantitative reverse transcription polymerase chain reaction (qRT-PCR) of selected drought-responsive miRNAs.

Name	Normalized Read Count		*p* Value	qRT-PCR		*p* Value
	Control	Stress		Control	Stress	
Root						
gma-miR319d	19.23	43.90	0.00	1.00	1.60 ± 1.16	0.63
ahy-miR398	471.98	11.77	0.03	1.00	0.51 ± 0.11	0.01
aau-miR396	107.62	17.07	0.00	1.00	0.27 ± 0.10	0.00
mtr-miR1507-3p	17.71	2.83	0.00	1.00	0.22 ± 0.07	0.00
aqc-miR166a	11.23	1.94	0.00	1.00	0.21 ± 0.03	0.00
bdi-miR159a-3p	5.26	0.98	0.00	1.00	0.54 ± 0.23	0.12
aly-miR166a-3p	1915.66	673.23	0.02	1.00	0.34 ± 0.12	0.01
nta-miR482a	121.66	37.75	0.01	1.00	0.86 ± 0.11	0.27
aly-miR396b-5p	59.07	12.52	0.03	1.00	1.32 ± 0.54	0.59
Leaf						
ahy-miR3512	7.43	0.96	0.00	1.00	0.67 ± 0.09	0.03
gma-miR5368	30.07	9.89	0.00	1.00	0.63 ± 0.18	0.11
han-miR3630-3p	16.40	5.89	0.00	1.00	0.78 ± 0.07	0.04
mtr-miR5294a	6.39	53.70	0.01	1.00	1.04 ± 0.12	0.76
gma-miR319d	103.05	605.32	0.00	1.00	1.76 ± 0.62	0.28
mtr-miR156g-3p	1.04	17.56	0.01	1.00	2.49 ± 0.21	0.00

**Table 4:** Predicted targets for drought-responsive miRNAs identified from *M. truncatula* genome databases.

miRNA Name	Target Accession	Expectation	UPE	Target Start	Target End	Inhibition	Target Description
han-miR3630-3p	Medtr0168s0060.1	2.5	18.13	35	56	Translation	zein-binding protein
han-miR3630-3p	Medtr0021s0360.1	3	16.35	3893	3913	Cleavage	phospholipid-transporting ATPase-like protein
han-miR3630-3p	Medtr1g015620.1	3	17.66	1832	1852	Cleavage	myosin heavy chain
han-miR3630-3p	Medtr2g039770.1	3	17.55	1184	1203	Cleavage	disease resistance protein (TIR-NBS-LRR class)
hbr-miR6173	Medtr8g009970.1	3	15.51	593	612	Cleavage	splicing factor 3A subunit 2
ahy-miR3512	Medtr8g063940.1	2	21.78	980	999	Cleavage	spermidine synthase
ahy-miR3512	Medtr3g058220.1	2.5	15.17	1732	1751	Cleavage	cytochrome P450 family 71 protein
ahy-miR3512	Medtr7g028160.1	3	13.87	1624	1643	Cleavage	TCP family transcription factor
aly-miR157a-3p	Medtr8g101880.1	3	15.82	23	42	Cleavage	ATP synthase G subunit family protein
mtr-miR5213-5p	Medtr3g025460.1	0	14.58	73	94	Cleavage	neutral/alkaline invertase
mtr-miR5213-5p	Medtr4g014580.1	1.5	13.06	121	142	Cleavage	TIR-NBS-LRR class disease resistance protein
mtr-miR1507-3p	Medtr7g091550.1	2	20.51	883	904	Cleavage	NBS-LRR disease resistance protein
mtr-miR1507-3p	Medtr7g078630.1	2	22.30	655	676	Cleavage	cysteine proteinase superfamily protein
mtr-miR1507-3p	Medtr5g015600.1	3	15.75	1400	1419	Cleavage	condensin-2 complex subunit G2, putative
nta-miR482a	Medtr6g463480.1	1	17.17	329	350	Cleavage	kinesin KIF2A-like protein
nta-miR482a	Medtr6g477950.1	1	13.93	39	60	Cleavage	B3 DNA-binding domain protein
nta-miR482a	Medtr7g088640.1	2	17.84	550	569	Cleavage	NBS-LRR type disease resistance protein
nta-miR482a	Medtr7g026400.1	3	14.25	1263	1283	Cleavage	response regulator receiver domain protein
nta-miR482a	Medtr5g099160.1	3	14.68	67	86	Cleavage	cation/calcium exchanger, putative

Notes: UPE: unpaired energy.

## Supplementary Materials

The following are available online at www.mdpi.com/2073-4425/8/4/119/s1. Figure S1: Melting curves of qRT-PCR products. Table S1: Members and expression abundance of known miRNAs from leaves of alfalfa treated with drought and irrigation. Table S2: Members and expression abundance of known miRNAs from roots of alfalfa treated with drought and irrigation. Table S3: Mature and precursor sequences of predicted miRNAs and the other information including reads length, reads number, GC contents and minimum free energy. Table S4: Members and expression abundance of drought-responsive miRNAs from roots of alfalfa treated with drought and irrigation. Table S5: Members and expression abundance of drought-responsive miRNAs from leaves of alfalfa treated with drought and irrigation. Table S6: Targets of drought-responsive miRNAs from leaves of alfalfa. Table S7: Targets of drought-responsive miRNAs from roots of alfalfa. Table S8: Sequences of the forward primers.
